# Exosomes form tunneling nanotubes (TUNTs) in the blood-brain barrier: a nano-anatomical perspective of barrier genesis

**DOI:** 10.3389/fnmol.2022.938315

**Published:** 2022-09-20

**Authors:** Shireen Mentor, David Fisher

**Affiliations:** ^1^Neurobiology Research Group, Department of Medical Biosciences, University of the Western Cape, Cape Town, South Africa; ^2^School of Health Professions, University of Missouri, Columbia, MO, United States

**Keywords:** exosomes, nanovesicles, tunneling nanotubes, blood-brain barrier, cell-to-cell communication

## Abstract

The blood-brain barrier (BBB) is a robust interface between the blood and the central nervous system. Barrier type endothelium is able to limit paracellular (PC) movement, relegating molecular flux to the transendothelial pathways of brain endothelial cells (BECs). It is, therefore, apparent that any leakage *via* the PC shunts would effectively nullify the regulation of molecular flux across the transcellular pathways. The application of higher-resolution scanning electron microscopy (HR-SEM) illuminates the heterogenous, morphological profile that exists on the surface of BEC membranes and the relationship between these ultrastructures during the molecular construction of the PC space between adjacent BECs. In this study developing BEC monolayers were grown on mixed, cellulose esters insert membranes in a bicameral system. BEC monolayers were fixed in 2.5% glutaraldehyde, hydrated, critically dried, and sputter-coated, for imaging utilizing HR-SEM. This study, for the first time, showed membrane-bound exosomes were attached to the plasma membrane surfaces of the BECs. The exosomes were characterized as small membrane-bound, nano-sized exosomes (30–300 nm). Based on their membrane morphology and anatomical structure, exosomes appear to possess two distinct functions, namely: paracrine secretion and nanotube construction between adjacent BECs, during *in vitro* barrier genesis. The HR-SEM micrographs in conjunction with the Tipifarnib inhibition of exosome formation, suggests that brain capillary endothelial exosomes play a prominent role in the bilateral signaling, which contribute to the regulation of the permeability of the BBB. Given that blood-brain barrier permeability has been implicated in the progression of many neurodegenerative pathologies, the role of these exosomes and TUNTs posits the capacity of these structures to exacerbate neuropathologies that implicate BBB permeability. These findings could lead to the development of novel treatment interventions and moreover, the characterization of BBB exosomes may be a reliable target for identifying therapeutic biomarkers in neurodegenerative disease. Conversely, the presence of BBB exosomes raises a critical enterprise to target the exosome-induced nanotubes as a vehicle for transferring therapeutic treatments across the BBB.

## Introduction

The blood-brain barrier (BBB) is a dynamic interface, which regulates the movement of substances and ions between the blood circulation and the brain’s microenvironment. It is comprised of highly specialized brain endothelial cells (BECs) which are tightly regulated by the interaction of intercellular tight junction (TJ) protein complexes. BECs, thus form the anatomical basis of the BBB. Although the BBB functions to maintain a well-regulated neuronal *milieu*, it is, however, also a major deterrent in the treatment of neurodegenerative disease. It, therefore, becomes naturally prudent to understand the physical functionality of BBB endothelium and to explore the possibility of molecular intervention. To date, the nanoscopic landscape of the BBB is unchartered territory. High-resolution scanning electron microscopy (HR-SEM) enables the observation of the ultrastructural, anatomical exploration of the BBB endothelium on a nanoscale. This study illuminates the complex, interactive nature of barrier genesis by describing the exosome landscape of the BEC as the nexus to the novel development of tunneling nanotubules (TUNTs) that facilitate BECs engagement and ultimately BBB formation. TUNTs are nanosized hollow membrane tubes which extend from cells and can interact with distal cells. The TUNT is a hovering apicolateral structure, with 50–200 nm diameters (Rustom et al., 2004). Its formation precedes the alignment of BEC lateral membrane domains. In some instances, this structure may be confused with filopodia. Filopodia are actin-rich structures, which present within basolateral domains of cells as a form of cell migration (Gurke et al., 2008).

Literature reports on the eukaryotic, nanosized extracellular vesicles (EVs) ranging between 40 and 1,000 nm in diameter and processing biomolecular contents, whereas TUNTs synergistically establish bi-directional, long-range cross-talk between cells (Muhammad and Farah, 2017). Moreover, intercellular BEC communication is essential to accomplish adjacent cellular alignment and molecular interaction within the proliferating environment of the brain capillaries. Recently, studies on the formation of nanotubes (NTs) in the developing *in vitro* BBB at high-resolution (HR) suggests it plays a key role in the alignment of TJ zones on the lateral membranes of BECs while forming the impermeable capillaries of the brain (Mentor and Fisher, 2021). This type of intercellular communication involves the formation of nano-sized exosomes (30–300 nm) which interact directly with target BEC membranes and thus its role has become a critical part of mediating intercellular communication (Purushothaman et al., 2016; Mentor and Fisher, 2021).

Over the last decade, exosomal regulation of direct cell-cell communication has emerged, particularly exocytosis of extracellular nanovesicles (NVs). Due to the heterogeneous nature of the NV, an interchangeable nomenclature relating to extracellular vesicles exist. In the extracellular *milieu*, NVs are referred to as exosomes, in cancer it is referred to as oncosomes and Golgi-derived NVs are derived as “gesicles” (Jaiswal and Sedger, 2019). We categorize vesicular structures of less than 1,000 nm in diameter as NVs, and for the purpose of continuity in nomenclature in this study, we will refer to these defined structures as exosomes.

**Figure 1:**
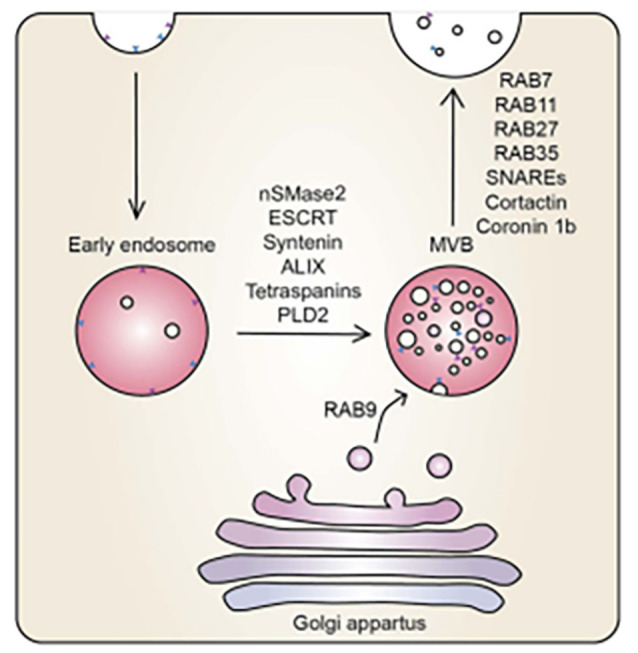
The mechanistic underpinning of exosome biogenesis. Vesicles from the Golgi apparatus fuse with the endosomes to form MVBs, regulated by an array of sphinmyelinases and GTPases (McAndrews and Kalluri, 2019).

Although, our study does not focus on the intracellular mechanisms of exosome formation, for the context of this study it is important to briefly summarise the process. Exosome production begins through the process of endocytosis with the formation of the endosome, normally encapsulating extracellular cargos at the plasma membrane. This early endosome matures into a late endosome. Thereafter, the late endosome matures into intraluminal vesicles (ILVs), which generate multi-vesicular bodies (MVBs). Exosome biogenesis is modulated by two mechanisms: (i) endosomal sorting complexes required for transport (ESCRT)-dependent or ESCRT independent mechanisms. MVBs are then transferred to the cell membrane, facilitated by proteins coactin, Rab, and GTPases to fuse with the plasma membrane, resulting in the exocytotic release of exosomes (Datta et al., 2018; Zhang et al., 2021; Figure 1).

During exosome biogenesis, the inward budding of MVBs of the Golgi apparatus is regulated by neutral sphingomyelinase 2 (nSMase2), ESCRT, syntenin, ALIX, tetraspanins, and phospholipase D2 (PLD2). The vesicles derived from the Golgi apparatus fuse with endosomes and are incorporated into MVBs. The MVBs fuse with the plasma membrane releasing their contents. Ras-related proteins (Rab; i.e., Rab7, Rab11, Rab27, Rab35) are proteins involved in GTPase-mediated signal transduction and protein transport, soluble N-ethylmaleimide-sensitive factor (NSF) attachment protein receptors (SNAREs), cortactin, and coronin 1b are involved in membrane docking of the vesicles (Datta et al., 2018; McAndrews and Kalluri, 2019). It is at this stage that we report on the convoluted BEC exosomal landscape and postulate functions that could contribute to the main function of the BEC, that of forming a robust regulatory barrier.

The high-resolution, microscopy-based visualization of the nanosized exosomes should be recognized as the gold standard for illuminating function between cells, and only when this is not possible should we resort to other molecular and fluorescence techniques to predict function. In this study, we report on the novel development of TUNTs from exosomes in BEC monolayers utilizing HR-SEM.

## Methodology

### Experimental model—the bicameral system

**Figure 2:**
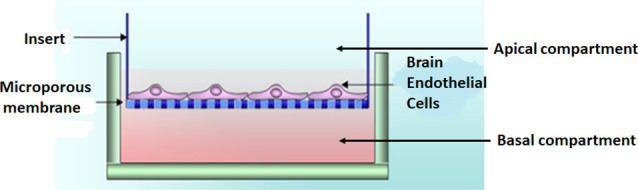
A bicameral system was used to establish a monolayer of bEnd5 BECs which formed the central component of the *in vitro* BBB model we used in this study. Adapted from Xu et al. (2013).

BECs (5 × 10^5^ cells/insert) were seeded on the upper side of mixed cellulose esters insert membranes, placed in a 24-well culture plate, creating a bicameral system. A bicameral system utilizes an insert membrane placed within a 24-well plate-BEC monolayer (i.e., bEnd5) grown on a mixed cellulose insert membrane (Merck, cat no PIHA1250, Germany; Figure 2). The cell monolayers were maintained in a standard culture medium (i.e., DMEM: F12), supplemented with 1% *Pen/Strep* and 10% Fetal Bovine Serum; 1% Sodium Pyruvate, 1% Non-essential amino acids) and were allowed 24 h to attain cell confluence before processing the biological sample for HR-SEM imaging.

### Chemical fixation

The confluent bEnd5 cells monolayers were fixed with 2.5% glutaraldehyde (BioChemika/Fluka-Sigma-Aldrich, Cat no. 49626) prepared in standard cultured buffer (i.e., phosphate-buffered saline solution (PBS); Faso et al., 1994; Fischer et al., 2012). Buffers and fixatives used in culture were maintained at pH 7.2 and at an osmolality, which equaled that of blood plasma (280–300 mOs/kg). Following a 1 h incubation period at room temperature (RT). The sample could be stored in a fixative at 4°C overnight. Thereafter, the specimen was washed in 1× PBS (devoid of glutaraldehyde) for 2 × 5 min (min) each. Thereafter, samples were washed twice in de-ionized H_2_O, each time for 5 min (Fischer et al., 2012).

### Dehydration

Biological specimens were removed from the de-ionized H_2_O and placed in a series of graded ethanol (EtOH) solutions: 50%, 70%, 90%, 95%, and twice in 100% EtOH for 10 min each. All EtOH solutions were prepared by diluting 99.9% absolute EtOH in de-ionized H_2_O v/v.

### Critical point drying

Critical point drying (CPD) was conducted for the phased drying of wet, delicate samples from liquid to gas form, by using liquid CO_2_, which functions as a “transitional fluid”. Since H_2_O is not miscible with CO_2_, thus, EtOH was used as the “intermediate fluid”/“dehydration fluid.” Following the dehydration of the fixed samples, it is required that the sample be dry before further processing could occur, this was performed using the Hitachi HCP-2 CPD (Figure 2). Evaporative drying of biological specimens could cause deformation and collapse from the native state of the sample. The deformities in the sample are often due to the surface tension of water, relative to evaporating air (Fischer et al., 2012). CPD was performed by immersing biological samples in liquid with a lower surface tension than air (i.e., carbon dioxide, CO_2_). Dehydrated samples were placed inside the CPD chamber and filled with liquid CO_2_. The chamber would not reach the critical point (CP) with inadequate liquid CO_2_. The temperature was set to 20°C for 15 min and then 38°C for 5 min until the critical pressure was reached (73 kg/cm^2^).

### Sputter coating

BEC monolayers were coated using a Gold: Palladium (Au: Pd) alloy, at ratios of (60:40). The sample was imaged using a Zeiss Auriga HR field-emission gun SEM, which was operated at an electron beam energy of 5 keV and a nominal working distance of 5 mm.

HR micrographs were collected utilizing an in-lens secondary electron detector, which allowed for HR image generation. The use of Au:Pd metal alloys as a routine coating method reduces damage to bioorganic samples (Zhou et al., 2006).

### Inhibiting exosome biogenesis—treatment with Tipifarnib

The chemical compound, Tipifarnib was employed to investigate its inhibitory effects on exosome biogenesis in bEnd5 cells as the anatomical basis of the BBB. Tipifarnib is medically known as farnesyl transferase (FTI), and functions by inhibiting exosome biogenesis and secretion *via* both ESCRT dependent and independent pathways (Datta et al., 2018). Tipifarnib effects were studied during BBB NT formation in order to determine its impact on BBB BEC interaction. BEC monolayers were treated with a range of 1–10 μM Tipifarnib in order to determine the point of exosome inhibition, and subsequent retardation of TUNT development.

### Effect of Tipifarnib on brain endothelial cell transendothelial electrical resistance

#### Transendothelial electrical resistance

Twenty-four hours (h) after seeding 5 × 10^5^ bEnd5 cells/insert/well on membrane inserts (Millipore, Darmstadt, Germany), in 24-well plates, they reached confluence and the cultured medium was replaced by selected concentrations of the exosome inhibitory agent: Tipifarnib at 1 μM, 5 μM, and 10 μM, relative to control samples. A humidified atmosphere of 5% CO_2_ at 37°C. Transendothelial electrical resistance (TEER) was carried out for 12 h, 24 h, and 48 h using a Millicell electrical resistance system (Millipore, Germany). Quantitative analysis of the recorded TEER readings employs the parameters of an appropriate equivalent circuit, which represents the electrical parameters across the *in vitro* BEC monolayer under investigation.

All morphological experiments utilized thousands of micrographs repeated over a 4-year period. All cell culture experiments were performed in triplicate or repeated a minimum of three times (for the detailed published protocols see Mentor et al., 2022a,b).

### Statistical analysis

The results are expressed as the mean ± standard error of the mean (SEM). Statistical calculations were performed using the One-Way ANOVA. Furthermore, the Tukey multiple comparsion test was utilized to compare the means between not normally ditributed, independent samples. Statistical significance was designated at *p* < 0.05.

## Results

### Phenotypic characteristics of membranous nanovesicles

The resolution of HR-SEM allows the observation of not only the plasma membrane products but also allows limited observation of the molecular make of these anatomical structures (Mentor et al., 2022b). Upon analysis of the surface topography of a BEC membranous surface, an array of exosomal nanovesicles were identified to accumulate on the plasmalemma of a bEnd5 cell.

### Non-TUNT forming exosomal interaction

**Figure 3:**
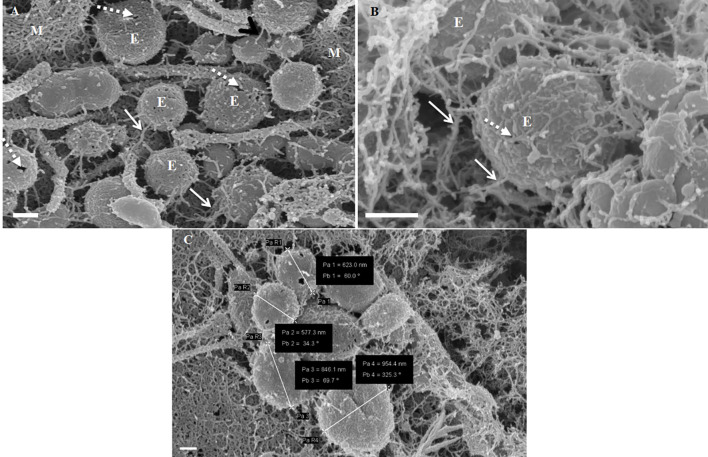
The formation of NTF-exosomes, on the BEC plasma membrane surface. **(A)** The HR-SEM micrograph depicts exosomes emanating from the bEnd5 plasmalemma. Scale bar = 200 nm. **(B)** A magnified version of NTF-exosomes accrued on the bEnd5 plasmalemma. The perforated, white arrows indicate the porous NTF-exosome membrane structures. Scale Bar = 300 nm. The “sticky” short filaments of the membrane-bound exosome anchoring to the BEC membrane surface, are indicated by white solid arrows. M denotes the plasmalemma surface, and E denotes Non-TUNT-Forming-exosomes. **(C)** A heterogenous population of measured, BEC exosomal nanovesicles. Scale bar = 200 nm.

The exosomes illustrated in Figure 3 were characteristically spherical, while some appeared as bi-vesicles during their formation. These exosomes were not conspicuous while cells were sparsely distributed on the membrane of the insert, but as continual cell division took place, leading to the close approximation of cells, these exosomes were increasingly formed on the plasma membranes of bEnd5 BECs. BEC exosomes were loosely attached to the extracellular apicolateral membrane surfaces with short adhesive “sticky” filamentous surface structures. These filamentous structures appeared to facilitate the exosome’s anchorage onto the parent cell membrane. Furthermore, the membrane of these exosomes had the same type of structure (molecular texture) as the parent plasma membrane to which they were attached, while their membrane structure was characteristically porous. Although we have not been able to report on the actual formation of these exosomes, within a closed cell culture environment, their origins are exclusive to the seeded bEnd5 BECs. This type of “non-TUNT forming” exosome (NTF-exosomes) was never observed to fuse forming tubular structures.

### Characteristics of TUNT forming exosomes

TUNT-forming exosomes (TF-exosomes) were observed to have a distinctly different texture to “non-TUNT forming” exosomes (NTF-exosomes). These TF-exosomes had characteristically smoother membranes but were also porous. It appears that these TF-exosomes were formed *via* a different packaging mechanism than NTF-exosomes. TF-exosomes appeared to form sequentially, but as distinctly separate vesicles. Furthermore, they were observed to form simultaneously on both apicolateral membranes of the juxtapose adjacent BECs. These TF-exosomes also have a propensity to form short, adhesive filaments anchoring them to the plasmalemma of the BEC.

**Figure 4:**
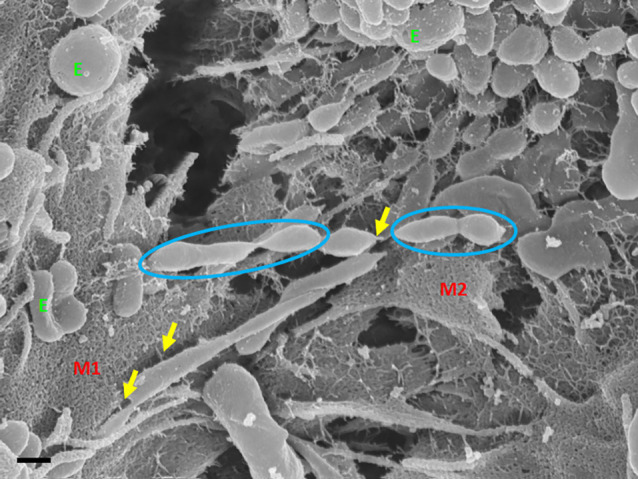
An HR-SEM microgragh illustrating the interaction of the distal ends of TUNT structures with target cell membranes and the interaction between individual TF-exosomes to form nanotubules. E denotes clustering TF-exosomes on the BEC membrane surface. Yellow arrows indicate interaction points between the TUNT and the BEC membrane and between individual exosomes. M1 denotes the cell membrane on which TUNT originates and M2 denotes the target cell membrane. The blue circles indicate exosome fusion during TUNT formation. Scale bar = 300 nm.

Exosome-exosome interaction is evident as the exosome-induced TUNT scaffolding network is formed at the apicolateral domain of the PC space (see Figure 4: yellow arrows). The HR-SEM micrograph illustrates two bEnd5 cell membrane leaflets juxtaposing each other (Figure 4) and the complex exosome microenvironment on the plasma membrane of these BECs. Prominent is the formation of TUNTs from the serial fusion of TF-exosomes. It is also important to note that the membrane of these exosomes was much smoother than the texture of both NTF-exosomes and the parent plasma membrane.

**Figure 5:**
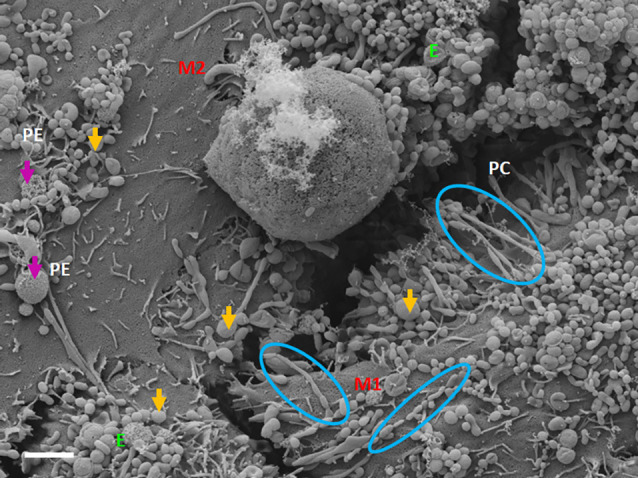
The expression of TF-exosomes on the bEnd5 plasmalemma is requisite for TUNT formation for the BECs monolayers. The above micrograph is an illustration of cell-cell communication promoted by the formation of two types of membrane-bound exosomes and the primordial stages of TUNT development and the lateral pervasion of TUNTs within the PC space following exosome accumulation on the surface of a bEnd5 cell. M1 denotes the cell membrane from which the TUNT originates and M2 denotes the target cell membrane, The purple arrows indicate porous NTF-exosomes (PE), and the orange arrows indicate TF-exosomes (E), which induce TUNT formation accumulating on BEC membrane surfaces, the blue circles indicate TF-exosome-induced TUNT formation. Scale bar = 2,000 nm.

**Figure 6:**
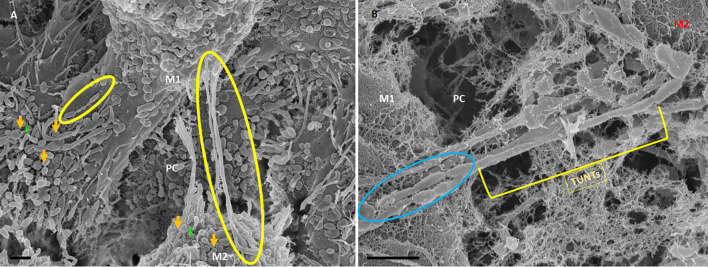
HR-SEM micrographs depicting bEnd5 exosome expression on a target cell. **(A)** An HR-SEM micrograph displaying TF-exosome-induced TUNT generation. Scale bar = 1,000 nm on a recipient cell M2. **(B)** A 200 nm micrograph of the open-ended TUNT formation, scale bar = 1,000 nm. E denotes clusters of exosome accumulation on the BEC membrane, the orange arrows indicate individual exosomes, M1 and M2 denote the porous membranes of the BEC from which NT originates (M1) and the target cell membrane (M2), the yellow circle and TUNT indicate tunneling nanotubes across PC, denotes the paracellular space. Scale bar 1,000 nm.

TF-exosomes appear to be able to fuse at their elliptical ends to promote the generation of TUNTs extensions along the apicolateral membrane borders and also across the PC space. TF-exosomal trafficking to form TUNTs occurs bi-directionally on both adjacent BECs. The TUNT emanates from the serial fusing of these TF-exosomes. The contact of the newly formed TUNTs extends from the apicolateral membranes of the BEC membrane across the PC space to form a TUNT-membrane interaction between the adjacent BECs (Figures 5, 6).

**Figure 7:**
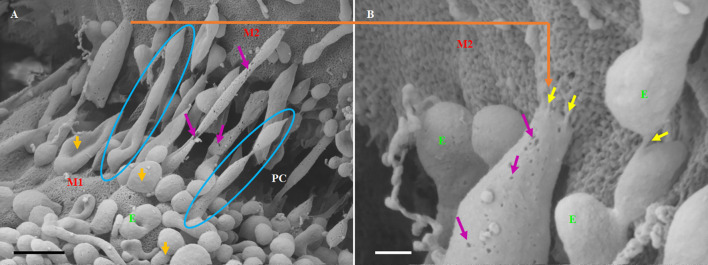
The formation of long-slender TUNTs is induced by the fusing of multiple TF-exosomes at their elliptical ends. **(A)** Illustrates TF-exosomes accrued to the BEC cell membrane and progressing into oblong-shaped, hollow tubes. Scale bar = 1,000 nm. **(B)** An enlarged area from “A” demonstrating the interaction of the TUNT “sticky” ends with the BEC membrane. Scale bar = 200 nm. E denotes TF-exosomes and M1 and M2 denote the porous membranes on which TUNTs originate (M1) and the target cell membrane (M2). PC denotes the paracellular space between M1 and M2. The blue circles indicate TUNT formation. The purple arrows indicate the pores on the TF-exosomal-induced TUNTs. The yellow arrows indicate the interaction between the distal extremities of TUNTs and the BEC membrane (Mentor and Fisher, 2021).

The distal extremities of these TUNTs display foot-like projections, which attach to the cell membrane on the target cell (M2—Figure 6A yellow circle). The orientation and direction of the extensions are signal-specific and appear to depend on the exosomal released chemical gradient that exists between adjacent BECs (M1—Figure 6A). Moreover, exosome-exosome interaction is evident as the exosome-induced TUNT scaffolding network is formed within the apicolateral domain of the PC space (see yellow arrows—Figure 7B below). The HR-SEM micrograph (Figure 7A below) illustrates the alignment of the bEnd5 cell exosome on M1 and shows its ability to fuse forming serially bi-, tri-, and multi- vesicular bodies, which fuse to form TUNTs across the PC space between adjacent BECs (see blue circles in Figure 7A below).

TUNTs typically extend across the PC spaces resembling intercellular, cross-bridges between adjacent BEC, which form a tunneling scaffold during BEC monolayer development, and possess a porous membrane filled with yet unidentified substances (Figures 7A,B). It must be pointed out that just as the TF-exosome’s membrane texture maintains continuity during the formation of the TUNT, displaying the smoother texture of the membrane as well as the generous distribution of pores (Figure 7B). By extension, there must also be continuity in the contents of these TF-exosomes as they fuse to form a tube. When cells make direct contact they form intimate physiological connections, which permit the sharing of signaling information between cells through these TUNTs (Jaiswal and Sedger, 2019).

### Characteristics of exosome-induced TUNT formation—a summary

**Figure 8:**
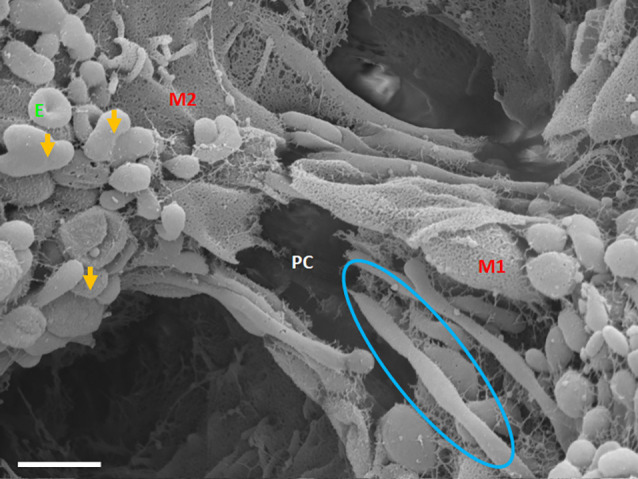
An HR-SEM micrograph exhibiting long, tunneling NTs (TUNTs) extending within the paracellular space between adjacent bEnd5 cells. TUNTs form extensive close-ended intercellular cross-bridges between cell membranes 1–2 (M1-M2). Notice the indented nodes along the length of the TUNT indicating the zones of fusion between TF-exosomes. M1 and M2 denote the porous membranes on which the TUNT originates (M1) and the target cell membrane (M2) and PC denotes the paracellular space, E denotes surface exosomes, the orange arrows denote individual Es, and the blue circle denotes primordial TUNT formation from E fusion. Scale bar = 1,000 nm.

TUNT development is an event that occurs, once a substantial amount of TF-exosomes accrue on a particular BEC. The TUNT extensions are bi-directional and are generated by the fusing of TF-exosomes into an intercellular nano-sized tube, hence the name tunneling nanotubule (TUNT). Fusing TF-exosomes begin to form primordial tunnels, which develop from a cell from which a mature TUNT originates and that traverses the PC space from M1 (on which TUNT originates) and aims to lodge itself onto M2, which denotes a target cell (M2) growing in close proximity (Figure 8 below).

### Primordial tunneling nanotubules

The cross-bridge TUNTs are both dynamic and transient phenomena, forming within 24–72 h *in vitro*. Figure 8 shows the TUNT extension from M1 towards M2. The close-ended tubular structure eventually becomes an open-ended tube once directly attached to the adjacent BEC cell (Figure 7B). These structures behave in a bi-directional manner, thus there is no dominant cell as both cells are working simultaneously in concert.

### Treatment with Tipifarnib

**Figure 9:**
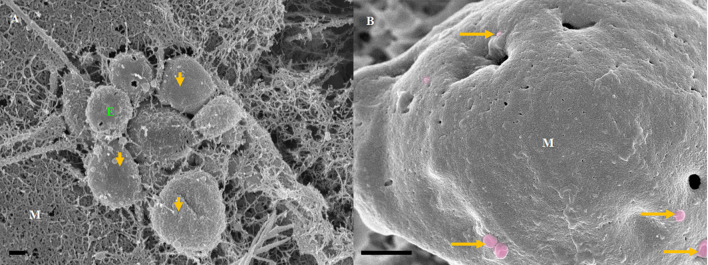
The transient emerging and fusion of the exosome on the plasma membrane of a BEC. **(A)** An untreated control BEC displaying exosome extrusion onto the BEC extracellular membrane surface. M denotes the porous, cell membrane. E denotes an exosome which is indicated by orange which may eventually fuse with the adjacent exosomes and give rise to a TUNT. Scale bar = 200 nm. **(B)** The Tipifarnib-treated BEC displays an observable decrease in exosome formation on the extracellular membrane surface upon treatment with 1 μM of Tipifarnib, indicated by the orange arrows. Scale bar = 2,000 nm.

Treatment with Tipifarnib resulted in the inhibition of the exosome formation. The compound has a direct effect on the rate of release of the exosome from the BEC (Figure 9B), relative to the control (Figure 9A).

**Figure 10:**
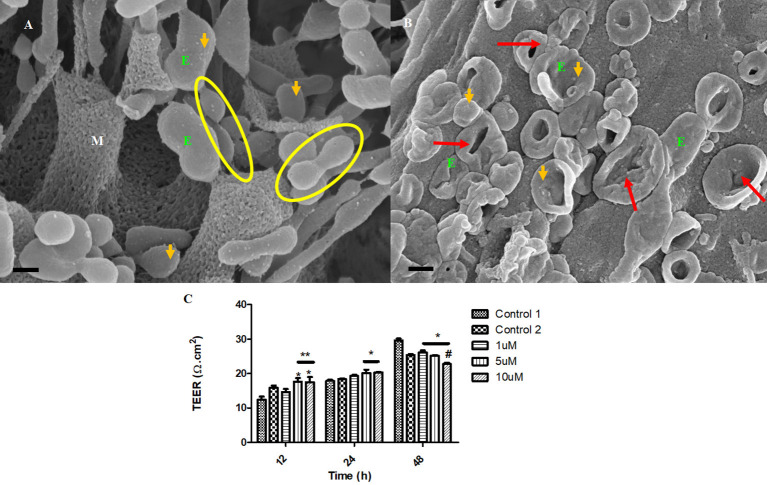
The accumulation of the exosomes on the BEC membrane surface and exosome-derived NT formation initiate BEC-cell communication. **(A)** The untreated control sample of BECs exhibiting spherical exosomes released onto the plasma membranes of the BEC. Scale bar = 200 nm. **(B)** The BECs were treated with 1 μm of Tipifarnib for 24 h. E denotes the exosome, indicated by the orange arrows. The red arrows indicate visible exosome collapse upon treatment with Tipifarnib. Scale bar = 300 nm. **(C)** A graphical representation of the effect of Tipifarnib on BEC (bEnd5) permeability upon 12 h, 24 h, and 48 h treatment. The single asterisk denotes statistically significant differences between the experimental sample and Control 1; the double asterisk denotes statistical differences between the experimental samples and Control 2; and the Hash denotes significant differences between 10 μM and 10 μM Tipifarnib.

The impetus of the exosome to generate tunneling nanotubes between adjacent BECs at a steady rate is highly inhibited upon treatment with Tipifarnib. Figure 10B displays the collapse in the exosome vesicular structure before it is able to engage neighboring exosomes to form TUNTs, compared to the control samples (Figure 10B), which appears to steadily form TUNTs upon TF-exosome fusion (Figure 10A). Figure 10C displays the ability of Tipifarnib to decrease TEER across the BEC monolayer over a 48 h period. At 12 h a significant increase in TEER was observed between the controls [i.e., C1 and the vehicle control (C2)] with 5–10 μM Tipifarnib (*P* < 0.001). At 24 h a significant increase in TEER was observed between the controls and 5–10 μM), however, at 48 h the continued exposure to Tipifarnib resulted in a significant decrease in TEER across the bEnd5 monolayer (*P* < 0.001).

## Discussion

The BBB is essentially a physical anatomical barrier that seals its paracellular (PC) spaces with TJs only allowing for molecular and ionic transendothelial transport *via* regulated channels and molecular transporters. Although this robust barrier is crucial for homeostatic regulation of the neuronal environment, it is also a fundamental obstacle in treating neurodegenerative diseases. It is, therefore, rational to understand the functional anatomical endothelium of the BBB and to investigate if molecular intervention may be utilized to modulate exosome function. Although the micro-anatomical and molecular structures have been well documented in the scientific literature, its nanoscopic landscape remains largely unexplored. Recently the scientific focus has been on how cells nanoscopically interact with each other under normal and pathological conditions. HR-SEM enables the observation of the nanoscopic anatomical complexity of the BBB endothelium. This study provides a glimpse of that complexity by describing the exosome landscape on the BEC and the novel development of TUNTs that facilitate the interaction of adjacent BECs.

### Nanovesicular exosomes

Exosomes are only found on bEnd5 cells once they grow closer to each other. The initially seeded bEnd5 cell displays a fairly denuded exosomal and NT landscape during the formation of the *in vitro* BEC monolayer. Once cells divided to the point where they were semi-confluent, exosomes were formed in abundance on the BEC surface. We could easily characterize two main types of exosomes, namely, those involved in the formation of TUNTs (TF-exosomes) and those that were not (NTF-exosomes). The formation of TUNTs from extracellular exosomes is novel for the endothelium of the BBB. We have not been able to currently find TF-exosomes in other epithelia or tissue. This may be that they are yet to be discovered or that TF-exosomes are peculiar to the endothelium of the BBB.

**Figure 11:**
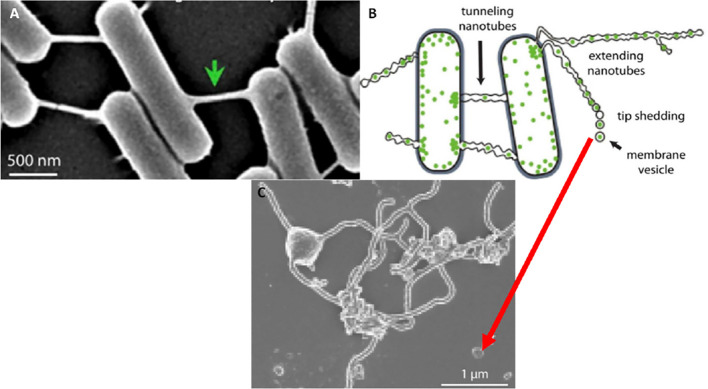
The formation of the tunneling nanotube and subsequent exosomes. **(A)** Intercellular nanotubes formed between adjacent bacterial cells. **(B)** An annotated diagram of exosome generation formed as a result of budding from existing bacterial NT extensions. **(C)** A micrograph exhibiting exosomal detachment from NT structures (Mentor et al., 2022a).

However, the documentation of extracellular vesicles (EVs) has been sighted in many bacterial species (Gözen and Dommersnes, 2020; Imachi et al., 2020). Here extending nanotubes (NTs) were found to connect bacteria, resulting in the interconnection of adjacent bacterial cells. The nanotubes were found to possess sequential segments, with a continuous luminal area (Tsafrir et al., 2001; Gözen and Dommersnes, 2020). Furthermore, when the distal ends of these bacterial TUNTs were not attached to an adjacent bacterium, the tips of these nano-sized tubular extensions would bud off, forming individual nano-sized exosomes. Thus, the connection between exosomes and TUNTs seems to be an evolutionary preserved feature of cell-cell communication namely, that the exosomes on both prokaryotic and eukaryotic species form from NT-induced exosome formation (Figure 11).

### Non-TUNT forming exosomes

This category of exosomes exhibits a porous and “sticky” topographical surface (Figure 3) and attaches itself to the plasma membrane on its apicolateral surface of the BEC. In the literature, the exosomes are reported to possess diameters ranging between 30 and 100 nm (György et al., 2011) and are denoted as microvesicles (MVs) when the EV’s diameter size ranges between 100 and 1,000 nm. The phenomenon of exosomes being extruded from cells is supported by work described in (György et al., 2011; Kimura et al., 2012) and has been reported to originate from endocytic vesicles, which have been exocytosed onto the cell surface (Figure 3). We report on the presence of exosomes once they have been extruded onto the surface of the BEC membrane. These exosomes range from 175 to 945 nm, averaging 750 nm, form through an intracellular process that allows for exosomes of functional and structural diversity to be extruded onto the surface of the cell (see Figure 1).

Mature cells, which are sparsely located do not communicate *via* exosomes, but in close proximity, they release paracrine factors as a means of cell-cell communication (Sahoo et al., 2011; Gartz and Strande, 2018). Upon the seeding of the BECs, there is an absence of the exosomes; their generation increases in colossal amounts upon the formation of denser BEC populations (Figure 3). Although we have not been able to report on the extrusion of these exosomes from the BEC plasma membrane, within a closed cell culture environment, its morphological origins are, by implication, the BEC itself.

### Role of non-TUNT forming exosomes

*In vivo*, these exosomes would be formed on the apicolateral surface of the endothelium, i.e., the adjacent luminal surface of the brain capillary. If these exosomes were not attached to the endothelium they would inadvertently be flushed away by the flowing blood, and play no role in the modulation of the PC chemotaxic events. However, their adhesive filamentous structures which anchor these NTF-exosomes to the surface of the BEC suggest that they are primarily to release their chemical contents into the PC space and play an important role in influencing the PC chemical *milieu*. The membrane porosity of the NTF-exosome supports this postulate, suggesting that the paracrine factors released from these exosomes play an important role in the fundamental process of sealing the PC space of the brain capillary endothelium (Sahoo et al., 2011; Gartz and Strande, 2018). At this stage, however, further research investigating exosomal contents is required to endorse its molecular validity.

### Exosome-forming TUNTs

BECs grown in close proximity were observed to form abundant closely approximated exosomes, which are attached to the cell’s plasmalemma. These exosomes are smooth in texture and distinctly different from the plasma membrane. They possess an ability to form adhesive “sticky” filaments which attach to the BEC surface membrane upon exocytosis (Figures 4–6). As these exosomes present on the surface of the cell membrane they interact with each other resulting in exosome-exosome interaction to fuse and elongate into intercellular TUNT orientated between apicolateral surfaces of adjacent BEC membranes (Figures 4, 5).

HR-SEM has allowed us to differentiate between these exosomes based on the molecular texture of their membranes. The TF-exosome membranes have a smoother molecular texture than that of the NTF-exosomes. This suggests that these exosomes are synthesized *via* a different cellular mechanism and that they are purposed for the fusion with each other to form TUNTs. Furthermore, their adhesive filaments, which anchor them to the parent BEC plasmalemma, endorse their role to form TUNTs that are involved in direct cell-cell signaling. These TF-exosomes also possess numerous pores, which may potentiate their involvement in chemotaxic events in the PC space.

As these TF-exosomes fuse to form TUNTs, their membranes continue to exhibit numerous pores. This suggests that TUNTs do not only bilaterally link adjacent BECs, but also play an important role in creating a very special chemical *milieu* within the PC space which modulates intercellular signaling, ensuring that BEC interaction is coherent, in sync, and aligned (Figures 6A,B).

This direct, bi-directional communication between adjacent BECs is crucial for the alignment of apicolateral zones of TJ interaction. TF-exosomes appear homogenous in both structure and vesiculate contents. The contents of the TUNTs by extension will also be homogenous. These TUNTs extend across the PC space linking the adjacent BECs together, ensuring that the same molecular signals are being sent to both cells simultaneously. This is essential to prepare the adjacent cells for physical and molecular TJ alignment. These TJs on juxtaposed lateral membranes of BECs can only seal the PC space if they physically attach to each other *via* their molecular extracellular membrane double loops. Should the zones be misaligned no sealing of the PC space can occur. Therefore, we postulate that cross-talk between adjacent cells *via* the exosome-induced TUNTs is crucial for the alignment of zones of TJs (Figures 3 and 4).

To date, it is uncertain as to whether the novelty of exosome-based TUNT formation is peculiar to the tight endothelia of brain capillaries or if they could be found in other epithelia, however, we are not aware of the reported occurrence of these types of TUNTs in any other tissue. The closes we come to approach this type of structure is when the reverse takes place in bacteria and the ends of TUNTs bud off as vesicular exosomes (Gözen and Dommersnes, 2020).

HR-SEM micrograghs display the snap-shot action of the exosomes, ability to influence the mediation of BEC-cell communication (Mentor and Fisher, 2021). Furthermore, these exosomal structures in other tissues are reported to be key role players in molecular signaling, by intercellular transferring of modulating information in major biological processes such as cell survival, apoptosis, immune disease, and neurological disease (Cheng et al., 2017). This study clearly demonstrates exosome generation as an event that takes precedence to direct cell-cell communication. Figure 4 suggests that exosomes are the first nanostructures that appear as BEC populations grow more closely to each other, and are thus a pre-requisite for and central to the transformation of the individual exosomes fusing into TUNT cross-bridges and, therefore, serve as a mediator of direct intercellular communication.

It is clear that TUNT forming exosomes are secreted in clusters, these clusters occur in close proximity, this suggests that there is a purposeful cellular process linked to TUNT formation. TUNTs appear to have distinctly different surface morphology in relation to the BEC membrane. Moreover, we postulate that non-TUNT-forming exosomes are distributed more evenly across the BEC membranes. We postulate that porous, NTF exosomes (Figure 3) are involved in the formation of chemical gradients/signaling between cells by way of paracrine communication. Conversely, TF exosomes fuse and amalgamate their contents upon the formation of tunneling tubes, during long-range, cell-to-cell communication.

The question must arise as to whether these mechanisms exist in the *in vivo* domain? When BECs develop into an *in vitro* monolayer they merely emulate as closely as they are allowed, to an *in vivo* approximation. We argue that the complexity in the development of an *in vitro* monolayer is not peculiar to the experimental conditions, but if anything reflects less complexity than the *in vivo* scenario. Nevertheless, the membrane interaction complexity seen during barrier genesis of the *in vitro* monolayer provides substantial confidence that it must reflect exosomal and TUNT processes of the *in vivo* brain capillary endothelium. The visualization of developing exosomes seen at HR in BECs, with TF-exosomes fusing to form TUNTs (Figures 4, 7, 8) suggests its ability to transfer its molecular amalgam between adjacent BECs during barrier genesis (Figures 7A,B; Mentor and Fisher, 2021).

### Tipifarnib effects on exosome biogenesis

Tipifarnib is a farnesyltransferase inhibitor, which acts by blocking the activity of the farnesyltransferase enzyme, ultimately preventing Ras from binding to the membrane, rendering it inactive. This drug has been reported to inhibit exosome formation *via* the endosomal-sorting complex required for transport (ESCRT)-dependent and ESCT-independent mechanistic pathways, that attenuates the production and secretion of the exosome, in conjunction with the down-regulation of Programmed death-ligand 1(PD-L1) expression on sub-unit sensitive exosome (Greenberg et al., 2022). Based on the research findings on BEC (bEnd5) cells Tipifarnib intervention results in the abrogation of BEC exosome generation (Figures 9A,B) which subsequently fails to fuse and form bi-vesicles, ultimately preventing the induction of the TUNT extension across the PC space (Figures 10A,B). Thus the interaction between the BECs becomes inactive. The morphological deformities are observed in the inability of BECs to form exosomes (Figure 9B) and in the collapsed exosomes (Figure 10B) and ultimately the failure to form TF exosomes. This data is supported by the permeability study, which indicates the time-dependent increase in permeability of a BEC bEnd5 monolayer upon exposure to Tipifarnib (Figure 10). It is fundamental for repetition and re-evaluation of this work to be carried out in both primary and human cell brain microvascular endothelial cell lines irrespective, there is no rationale that suggests these convoluted and novel structures are peculiar to immortalized mouse brain endothelial cell line (bEnd5) and that they do not exist *in vivo*.

The data provides a case-in-point that is meaningful in that it strongly advocates that BECs can be regulated and molecular interventions that regulate these nanoscopic structures can be used to modulate BBB permeability. Furthermore, this implies that molecular intervention remains a plausible avenue for neurodegenerative treatment interventions.

### The role of exosomal delivery in the propagation of neurodegeneration

It is very clear that exosomes are key in establishing the primary functional role of the BBB (i.e., barrier genesis). The impermeability of the BBB is crucial to the homeostasis of the neuronal environment and also ensures that toxic substances that cause neurodegeneration are transported out of the brain, while toxic blood-borne substances are kept out of the brain.

Exosomes from metastatic cancers (i.e., oncosomes) have been reported to utilize the oncosome secretions from the site of the primary tumor into the blood to prepare distal anatomical sites for metastatic access of cancer cells (Jaiswal and Sedger, 2019). Given our current understanding of the crucial role that TF-exosomes play in the process of barrier genesis at the BBB, it is patently clear to comprehend the BBB endothelium’s vulnerability to manipulation *via* extraneous exosomes, which could be designed to increase permeability and access to metastatic cancer cells and other predatory blood-borne cells. Furthermore, it is well known that exosomes are released in colossal amounts and may be involved in both an immune response to prime tumor progression and survival *via* tumoral angiogenesis (Tamkovich et al., 2016; Zhang et al., 2021). This may have far-reaching clinical implications once we understand the effects of extraneous exosomal-based signaling mechanisms on the BBB.

Parkinson’s disease (PD) and multiple sclerosis (MS) are all neurodegenerative diseases characterized by BBB disruption caused by an increase in transendothelial permeability (Sharif et al., 2018). This allows for neurotoxic components of the plasma to enter the brain parenchyma, which in turn exacerbates neurodegeneration. In the AD mouse model increased BBB permeability was reported before any appearance of cognitive dysfunction and amyloid plaques (Maki Ujiie et al., 2003). This observation together with increased BBB permeability observed in neurodegenerative disease contributes to neuronal dysfunction and death and forms the basis of the “vascular model of neurodegeneration”. This model is supported by a study by (Dickstein et al., 2006) who reported that immunization of Alzheimer’s disease mouse strains with antibodies against amyloid beta peptides could restore BBB integrity by decreasing the permeability across the BBB. It is, therefore, clear that the relationship between BBB permeability and neurodegeneration may be causative in AD and may exacerbate existing neurodegeneration in PD and MS. Our studies indicate that TUNTs and exosomes are both peculiar and crucial to barrier genesis across monolayers of BECs. It is, therefore, highly plausible that any compromise to the normal formation of exosomes or TUNTs in the formation of the BBB endothelium may compromise its permeability and contribute or indeed be causative in neurodegenerative disease.

Furthermore, there is increasing evidence that shows exosomal involvement in the process of AD by its ability to spread amyloid-beta (Aβ) and hyperphosphorylated tau in the brain leading to neuronal damage (Takahashi et al., 2002; Baker et al., 2016; Sardar Sinha et al., 2018; Kalluri and LeBleu, 2020; Zhang et al., 2021). However, although it is known that the exosome is implicated in AD progression the exact mechanism of the pathological process remains to be investigated. We hereby postulate that one of the mechanisms whereby increased BBB permeability is a hallmark of AD occurs *via* an exosomal process that interferes with the normal process of TF exosomal formation of TUNTs during brain endothelial barrier genesis. We hypothetically pose that pathologies that compromise these TUNTs may play a role in AD, however, we stress that this postulated hypothesis is not established experimentally and must be tested in future research studies.

Moreover, exosome exists in both CSF, blood, urine, and bodily fluids and thus can be used as a diagnostic marker or as a target for drug delivery (Zhang et al., 2021). Exosome-like NVs have been reported to act in facilitating essential processes, namely: apoptosis, invasion, angiogenesis, resistance to therapy, and endothelial cell invasion (Peinado et al., 2011, 2012; Kharaziha et al., 2012; Thompson et al., 2013; Purushothaman et al., 2016) and BEC-cell interaction during barrier genesis (Mentor and Fisher, 2021). Since the exosome exhibits the propensity to mediate intercellular signaling and interact with the cell surface (i.e tumor derived proteins and nucleic acids) alludes to its ability to play a critical role in promoting disease progression.

A study by Alvarez-Erviti et al. (2011) engineered the exosome with siRNA load to target the CNS cells and silence Alzheimer’s disease β secretase enzyme-1 (BACE1), which caused a decrease in Aβ_1–42_. However, at this stage, effective exosome loading to target specific cells remains to be elucidated.

## Conclusion

Unlike in bacterium and archaea which bud off NT ends to produce exosomal vesicles, during BEC monolayer development TF-exosomes fuse to form TUNTs, with continuous lumen to ingeniously directly signal adjacent BECs during BBB genesis with the same molecular signals. We postulate that this direct signaling is crucial to aligning the apicolateral membranes of adjacent BECs so that their TJ zones could interact and seal off the PC space. Furthermore, the BECs generate two types of exosomes: (i) NTF-exosomes that have the propensity to mediate cell-cell interaction and function by creating a chemo attractant gradient within the PC space; and (ii) TF-exosomes that are formed repetitively and asynchronously, which fuse with each other to form TUNTs during BEC barrier genesis.

Based on the snap-shot development of BEC TUNT formation, we can infer that exosomes have signaling potential *via* the intercellular, cross-bridge TUNT formation in BECs, which could be implicated in the spread of molecular signaling during tissue formation and in the progression of pathology. Moreover, the exosome displays the potential to play a critical role in drug delivery across the BBB. At this stage mechanism of exosome extrusion from the BEC and its ability to transform into TUNTs crucial for functional BBB genesis, suggest its propensity as a promising target for disease intervention.

## Data Availability Statement

The original contributions presented in the study are included in the article, further inquiries can be directed to the corresponding author.

## Author Contributions

SM shares first authorship. DF shares last authorship. All authors contributed to the article and approved the submitted version.

## Funding

This research received no external funding. Funding was received from the University of the Western Cape (UWC) Senate funds.

## Conflict of Interest

The authors declare that the research was conducted in the absence of any commercial or financial relationships that could be construed as a potential conflict of interest.

## Publisher’s Note

All claims expressed in this article are solely those of the authors and do not necessarily represent those of their affiliated organizations, or those of the publisher, the editors and the reviewers. Any product that may be evaluated in this article, or claim that may be made by its manufacturer, is not guaranteed or endorsed by the publisher.
